# A Scoping Review on Bioethics Challenges of Conducting Clinical Research in Patients with Traumatic Brain Injury: Revisiting the Informed Consent Process

**DOI:** 10.3390/neurosci7030051

**Published:** 2026-04-27

**Authors:** Ayman El-Menyar, Naushad Ahmad Khan, Hassan Al-Thani

**Affiliations:** 1 Clinical Research, Trauma & Vascular Surgery, Hamad Medical Corporation, Doha P.O. Box 3050, Qatar; nkhan13@hamad.qa; 2 Clinical Medicine, Weill Cornell Medical College, Doha P.O. Box 24144, Qatar; 3 Trauma Surgery Section, Hamad Medical Corporation, Doha P.O. Box 3050, Qatar; halthani@hamad.qa

**Keywords:** emergency research, traumatic brain injury, informed consent, deferred, proxy, ethics

## Abstract

**Background**: Conducting research in emergency departments and critical care units is crucial for improving patient management through evidence-based practices. Healthcare professionals and researchers in the field of traumatic brain injury (TBI) have a moral and legal obligation to inform patients before conducting any diagnostic test or therapy as part of a clinical study. However, challenges and barriers to conducting research in these high-pressure environments must be acknowledged. Shall the pathway to obtain informed consent in TBI-related research be revisited? We sought to map literature, identify gaps, and clarify the bioethics that should be followed in TBI-related research. **Methods**: A Scoping review was conducted to identify the obstacles and challenges investigators encounter in clinical and translational TBI research, with a specific emphasis on informed consent and regulatory impediments that often serve as bottlenecks or rate-limiting steps for participant enrollment and overall study success. This review used google scholar and Midline from inception to 2025. **Results**: Patients with TBI or their surrogates may be unable to provide informed consent within limited therapeutic windows. Despite international regulations and national laws, restrictions on obtaining consent are often criticized as ambiguous in certain situations. Furthermore, the fast-paced, emotionally charged atmosphere in emergency settings poses a risk of delaying crucial research interventions. There are accepted alternatives to informed consent, such as proxy consent, deferred consent, exceptions from consent, and waivers of consent, which are ethically and socially acceptable and compliant with regulations. However, these alternatives are underutilized or may be abused in some cases. **Conclusions**: This review calls for clarifying and modifying arbitrary regulatory restrictions on research and streamlining the Common Rule. Scientists should also share their innovative solutions to strike a balance between ethical considerations and the minimization of research barriers.

## 1. Introduction

Clinical studies have resulted in remarkable advancements in preventing, detecting, and treating traumatic brain injury (TBI). The decline in TBI-related fatalities, which have dropped by approximately 30% in the last two decades, is a testament to the impact of these studies [1,2,3]. Despite advances, TBI remains the leading cause of disabilities and death among young people worldwide and is projected to remain among the top three leading causes of injury-related deaths [4]. Approximately 50 to 60 million individuals experience a TBI each year, resulting in an annual cost of around 400 billion USD to the global economy [5]. TBI is now recognized as a chronic disorder with long-term consequences, including an increased chance of acquiring neurodegenerative diseases later in life [6].

When conducting research on human subjects, it is crucial to closely adhere to ethical guidelines and regulations to deliver optimal, clinically appropriate treatment to patients and gather more precise information to inform future therapeutic recommendations [7,8]. The concept of “informed consent” remains one of the well-established and fundamental principles underpinning clinical and translational research since it establishes a physician’s fiduciary obligation to the patient and guides the course of the therapeutic relationship [9]. Patients have the right to be informed about planned studies and to make an informed decision regarding their participation [10].

From a legal standpoint, informed consent ensures accountability and a shared sense of responsibility between healthcare providers and patients. At the same time, from an ethical viewpoint, it upholds patients’ autonomy, privacy, welfare, and choices [11,12]. In TBI research, this process becomes highly complex and context-dependent, thereby defying easy categorization [13]. Obtaining informed consent is particularly challenging in emergency settings, where patients may have impaired cognition, be sedated or intubated, causing their decision-making abilities to be compromised, and proxies who may not be available or unable to provide consent, in addition to being in a stressful situation [14,15,16,17]. These ethical, logistical, and regulatory challenges necessitate a nuanced, context-sensitive approach to informed consent in TBI and trauma research in emergency settings. The sudden and unpredictable nature of trauma requires healthcare professionals to make rapid decisions and take immediate actions in the patient’s best medical interests. These challenges contribute to the variability in practices across emergency medicine research, particularly in obtaining patient consent. As a result, the necessity and mechanism for securing research authorization remain debatable when informed consent is unattainable.

## 2. Search Strategy and Data Synthesis

This review aimed to explore the ethical and regulatory challenges associated with conducting clinical and translational research in patients with TBI. The literature was examined using a scoping review approach with thematic synthesis to map the existing evidence and conceptual frameworks related to informed consent, regulatory oversight, and ethical considerations in emergency research. Relevant publications were identified through searches of electronic databases including MEDLINE (via PubMed) and Google Scholar from inception to 2025. The search strategy combined controlled vocabulary and free-text terms related to TBI, emergency research, and informed consent. The primary keywords included: “traumatic brain injury,” “TBI,” “neurotrauma,” “emergency research,” “clinical research ethics,” “informed consent,” “proxy consent,” “deferred consent,” “waiver of consent,” and “exception from informed consent (EFIC).” The findings were synthesized using a thematic approach, allowing the identification and organization of key domains such as informed consent challenges in emergency settings, alternative consent models (e.g., proxy consent, deferred consent, and exception from informed consent), ethical considerations involving vulnerable populations, and regulatory challenges associated with institutional review boards. The synthesis aimed to provide a comprehensive overview of current ethical frameworks and identify gaps that may influence the design and implementation of future TBI research.

We sought to map literature, identify gaps, and clarify the bioethics that should be followed in TBI-related research. This review involved all kinds of studies, review articles, relevant protocols and guidelines published in English.

The review excluded abstracts, case reports, conference proceedings, non-English literature and research in pediatrics. All relevant data were collected by two independent authors (AE and NK). Quality assessment and statistical analysis were not required apart from tables and illustrations; however, descriptive summaries and recommendation were performed. Preferred Reporting Items for Systematic reviews and Meta-Analyses extension for Scoping Reviews (PRISMA-ScR) Checklist was followed (Supplementary Materials File S1).

## 3. Discussion

### 3.1. Balancing Science and Ethical Complexities in TBI Research

In patients with severe brain injury, research in disorders of consciousness (DoC) presents complex ethical and regulatory challenges [17,18]. To overcome these complexities, further investigation is warranted within the realm of clinical and translational research [19]. Notably, following acute brain injury, secondary insults contribute to morbidity and mortality. Therefore, translational and clinical studies focusing on neuroprotection and prevention of secondary damage are crucial. However, these efforts demand increased ethical vigilance and nuanced regulatory frameworks to balance innovation with patient safety, particularly given the socio-behavioral and neuro-ethical dilemmas common in TBI research [20].

The absence of standardized methods for assessing decisional competence increases the risk of enrolling participants without adequate informed consent [6,14,19]. While the majority of TBI patients eventually regain decision-making capacity, there are still scenarios in which a waiver or surrogate consent may be required, or at least temporarily, at the initial stages of the injury. Additionally, TBI research may raise complex ethical issues within the evolving field of neuroethics [21,22].

**Figure 1:**
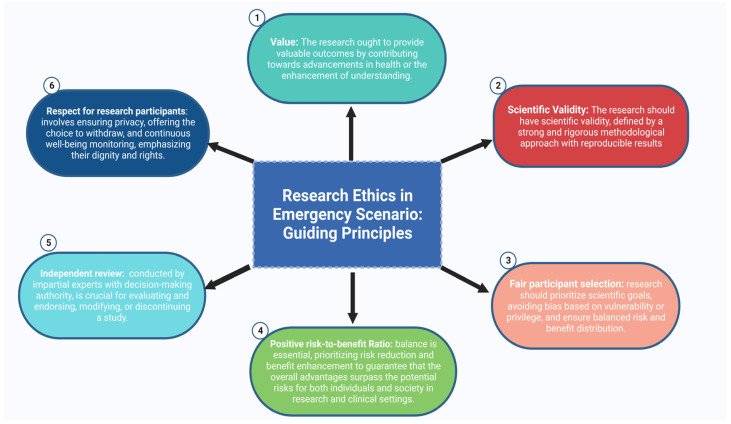
Established ethical standards.

There are several challenges in clinical and translational emergency research [23,24]. Debates persist regarding the goals and optimal implementation of informed consent, its adaptation for vulnerable populations, and the validity of alternatives such as broad or electronic consent [25]. Therefore, preserving participant autonomy via substituted consent is essential in TBI research. However, it alone cannot ensure the ethical conduct of the trial. Emmanuel et al. [26] put forward a set of additional ethical criteria for assessing clinical research within this specific domain, grounded in well-established ethical standards, as illustrated in Figure 1. Adherence to ethical standards is crucial for the implementation and conduct of responsible clinical and translational research, ensuring the well-being, respect, and rights of participants while advancing scientific investigation. Collaborative efforts among clinicians, neuroscientists, and neuroethicists [14,18,22,27] can help address knowledge gaps in TBI research by integrating input from families and key stakeholders. Such collaboration fosters ethically sound trial designs, enhances transparency, and safeguards participant well-being, ultimately strengthening the ethical and scientific foundations of TBI research and its translational impact.

### 3.2. Unresolved Ethical Challenges in the Study of Severe TBI

Despite advances in neuroscience, preclinical and clinical translation remain limited due to inadequate models, heterogeneous populations, and methodological gaps, reinforcing the need for rigorous preclinical studies before first-in-human trials [28,29,30]. Voluntary informed consent is fundamental in translational research, but in TBI patients with impaired cognition or communication, enhanced protections are required to safeguard autonomy and ethical participation [31].

Ideally, obtaining informed consent in a language the study subjects understand is imperative, while avoiding intricate medical terminology. However, the complexity of clinical and translational research, especially in TBI patients with acute neurological injuries or cognitive impairments, presents significant challenges in obtaining voluntary informed consent. Bioethicists hold diverse perspectives on whether to involve vulnerable populations in first-in-man translational research [32]. The overwhelming consensus among these experts is to recommend excluding such populations from studies that involve risks above the minimal level [33]. Nonetheless, continuous exclusion would prevent these people from benefiting from newly developed therapies. Ongoing clinical research involving TBI patients has revealed numerous areas where legal and ethical dilemmas remain unaddressed in the context of research with severe TBI. The authors emphasize that ethical concerns in translational research on TBI persist and encompass various issues discussed in the following sections.

### 3.3. Preserving TBI Patient Autonomy in Clinical and Translational Studies

The doctrine of informed consent has encouraged patients to actively engage in their health decision-making processes [34,35,36]. Researchers may encounter conflicts when balancing their duties with their obligations to society and the broader scientific community. The Declaration of Helsinki emphasizes that although medical research aims to advance our knowledge, it should never precede the rights and interests of individual research participants [34]. Therefore, clinical studies pose a challenge; nevertheless, it is crucial to develop therapies for this group as they face treatment options and high rates of mortality and long-term disabilities [37].

The ethical dilemma facing researchers is their need to study individuals with DoC, which may occur after severe brain trauma, to gain insights into their cognitive abilities and involve these subjects, when possible, in the informed consent process [38,39]. Moreover, some experts have pointed out that individuals with DoC have often been excluded from research [40]. However, this exclusion contradicts the principles of justice ethos outlined in the Belmont Report [40]. Instead, they argue that there is a need to include incapacitated individuals in research, while ensuring that ethical considerations and careful evaluations of foreseeable risks and benefits are met [40].

**Figure 2:**
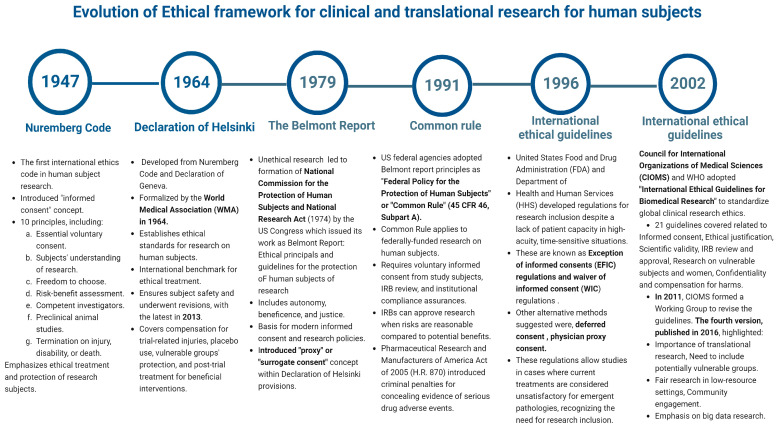
A timeline evolution of summarizing the ethical framework for research involving human subjects.

Consequently, instead of outright excluding incapacitated individuals with DoC from clinical and translational studies due to their inability to provide direct consent, investigators and healthcare professionals collaborated with ethicists to develop research designs that strike a balance between respecting patient autonomy and promoting beneficence and ensuring justice and avoiding harm while still facilitating research inclusion [40]. Some of these approaches include (i) having a surrogate or proxy decision-maker provide consent on behalf of the participant, (ii) using an exception from informed consent (EFIC), which allows for the waiver of informed consent, (iii) deferred consent with subsequent debriefing, (iv) utilizing a model that acts as a substitute for formal consent [14,19]. These different paradigms can be integrated in various ways into a flexible, adaptive, or multi-modal system of informed consent, particularly in a clinical trial involving individuals with TBI, where variations in participant capacity or the availability of surrogates are expected. Each model has its own set of ethical and operational advantages and challenges. A timeline of the evolution of the ethical framework for research involving human subjects is outlined in Figure 2.

### 3.4. Time-Sensitive Nature of TBI Research

Studies conducted in both experimental and clinical settings have demonstrated that pathophysiological cascades are triggered within minutes to hours after severe injury [41]. As a result, therapy windows are limited. Moreover, specific biomarkers typically reach their peak in the blood within a few hours and should be obtained early post-injury. Therefore, therapy or blood monitoring must be dealt with within a limited period to test the research studies’ hypotheses and objectives. Given that patients with severe TBI lose consciousness quickly and often remain that way for extended or unpredictable periods, obtaining direct consent from them is not possible. Delays in the delivery of experimental therapies are a possible explanation for the lack of progress in clinical and translational research on TBI [42].

It is critical to consider the unexpected nature of neurological recovery and the resulting memory loss in studies with individuals with TBI. It is essential to ascertain a patient’s competence to provide informed consent, as their cognitive state may change. Patients must be cognizant and able to communicate effectively to express their sense of self and provide informed consent. However, neuroscientists have no consensus on what defines consciousness [43]. However, in TBI clinical trials, the time windows for patient enrollment are usually determined by practical and logistical considerations rather than scientific evidence. Most studies use an 8 h window, some use 4 h, and a few allow enrollment up to 12 or 24 h after injury [44]. However, treatment initiation is often delayed until the last hour of the time window because informed consent from a legally acceptable representative is required. This requires the availability of legally authorized representatives (LARs) and next of Kin (NOKs) who are fully informed and given enough time to make a decision. The Declaration of Helsinki permits proxy consent from a third party (like a family member or legal representative) for individuals with mental or cognitive impairments [45,46]. However, obtaining proxy permission in emergencies poses challenges, particularly when patients are brought alone or when their attendants/relatives are emotionally unprepared to make rapid decisions. In situations like these, when the patient lacks decisional capacity and their family members are not open to the idea of participating in research due to the distress caused by their relatives’ admission to the ICU, there is a risk of missing out on research opportunities if consent cannot be obtained promptly.

Moreover, in some cases (moderate- to severe TBI), even obtaining consent from a surrogate decision-maker is not possible, as they are not readily available during the early hours. This often happens to workers (expatriates) who are working without family away from their home countries. If the surrogate cannot be found promptly, waiting for their arrival could result in losing the prospective patient within the time specified in the study protocol. In a study of 332 patients with severe trauma conducted by Wright et al., it was shown that only 28% of patients arrived at the hospital accompanied by their next of kin, highlighting the difficulty of obtaining proxy approval from next of kin, particularly when time limitations for emergency research are constrained, even in facilities with speedy referral durations [47].

Prior research has reported that eliminating the need for consent led to a higher enrollment rate and reduced the time to treatment by approximately 45 min, demonstrating a contradiction between the urgency to begin treatment and the time required for consent processes. Similarly, in a septic shock trial, the investigators were unable to contact the authorized family for 74% of patients during the inclusion period, and these patients were recruited under a consent waiver [48]. Similarly, the deliberate exclusion of TBI patients due to the difficulty of obtaining their consent may introduce bias into the results if the characteristics of those omitted (or who do not consent) differ significantly from those included in the research. Furthermore, it has been posited that when patients are excluded from the analysis due to severe illness or injury, subsequent refusal of consent or death before obtaining consent, despite their initial inclusion and completion of study procedures, may also lead to the introduction of substantial bias into the findings [49].

### 3.5. Alternate Consent Procedures in TBI Research

Healthcare providers and researchers are ethically and legally obligated to provide patients with information before undertaking diagnostic tests or therapy treatments in clinical trials. The lack of clarity and the restrictive nature of international norms and national legislation on this topic have been debated. Although approved alternatives exist, they are not often used, despite their ethical validity, social acceptability, and compliance with the rules. The proper use of these alternative consent methods holds promise for improving the efficacy and quality of future TBI research.

- Exception from Informed Consent (EFIC) and waiver of informed consent (WIC)

The United States Food and Drug Administration (FDA), in conjunction with the Department of Health and Human Services (HHS), acknowledged the need to include patients with emergency conditions in research, even if they were unable to give their consent owing to their critical condition and time constraints. Consequently, the EFIC regulatory pathway for drug and device research (21 CFR 50.24) [50] and the regulations on the waiver of informed consent (45 CFR 46.101) [51] for studies comparing standard-of-care treatments were established. Before these frameworks, potential participants in emergency research were often excluded because their acuity prevented them from making meaningful research decisions and from timely contact with LARs.

Under EFIC/WIC provisions, study information must be conveyed to patients or proxies as soon as possible, and any decision to decline further participation or data use must be respected. These mechanisms are particularly relevant when urgent or prolonged interventions preclude obtaining prospective consent. Numerous articles have been published regarding the use of EFIC, including considerations and public opinions on research conducted without consent, as well as the role of surrogate decision-makers in research-related processes [52,53]. Furthermore, numerous articles have explored the complexities and additional challenges that arise when implementing these regulations within a research context [54,55]. However, conducting research without prospective informed consent remains ethically sensitive, as it may impose additional risks on already vulnerable individuals, especially when the efficacy of the intervention has not been established. The EFIC concept raises ethical concerns in randomized controlled trials (RCTs), demanding rigorous adherence to the research protocol, particularly in life-threatening situations such as severe TBI [55]. Methodological limitations, including challenges in generalizability and cross-study comparisons, further restrict their broader applications [56].

Despite their potential, EFIC and WIC have been used sparingly over the last 25 years. A review of 28 acute care studies published between 1996 and 2018 found only 24 EFIC studies and 4 WIC studies, highlighting the limited use of these regulations [45]. To evaluate healthcare providers’ attitudes and opinions on enrolling patients in research without consent, one study from the Middle East found that only <20% of ED healthcare providers agreed to enroll their family and/or community members in emergency research without consent. Another study in the USA found that 31% of EMS providers agreed to enroll patients without consent. These findings may reflect limited experience in scientific research and a lack of confidence in conducting and facilitating emergency research [57,58].

In recognition of the above evidence and challenges, EFIC and WIC are rarely implemented in TBI research in practice, owing to the intricate ethical, logistical, and regulatory challenges inherent to this population [45]. Beyond the barriers previously discussed, additional constraints include the heterogeneity of injury severity, the fluctuating nature of cognitive recovery, and the difficulty of identifying eligible patients within the critical early time window for intervention.

At Hamad Trauma Center, deferred and NOK consent serve as a functional equivalent to EFIC in several studies. In the prehospital tranexamic acid study, 163 of 204 trauma patients (79.9%) were enrolled through deferred or NOK consent; of them, 106 (65%) completed the delayed informed consent once capable, while 41 (20.1%) provided consent on admission [59]. Similarly, in the BBTBBT clinical trial (the use of beta-blocker in TBI patients), 238 of 437 TBI patients (54.5%) were enrolled through deferred or NOK, with 171 (72%) subsequently able to sign the delayed consent, and 199 (45.5%) were consented on admission [60]. The reason for using deferred consent was the time window for blood test and medications, which should be administered within 24 h post-injury. We closely followed the patients even after transferring them to other facilities (e.g., rehabilitation centers) to ensure they could sign the delayed consent. The deferred consent should be signed by two physicians, one of whom should not be part of the study team. These studies were regularly supervised by the DSMB and by IRB auditing visits. These experiences highlight that although EFIC and WIC are not formally applied, comparable ethical mechanisms are effectively practiced, enabling the inclusion of critically ill patients, emphasizing the need for structured EFIC adoption in future trauma and neurotrauma research.

Currently, no established guidelines exist for reporting EFIC or WIC in their peer-reviewed publications. While some researchers outline the logistical requirements for implementing these methods, many omit ethical justifications or explanations for EFIC that are necessary to address the research question. Authors should explicitly describe the rationale for the EFIC/WIC and how patients or LARs are informed. Furthermore, the study should provide a comprehensive description of potential challenges that may arise after participant enrollment, including the number of subjects or their LARs who may refuse to consent to continued participation or withdraw from the research. Including these details would enhance transparency and strengthen public understanding.

Implementing EFIC trials also involves seeking input from community members who may participate in the study [61]. Researchers must ensure that the community is aware of the research, its benefits, and the option to decline participation [62]. By involving community members, researchers can assess their perceptions of the proposed research activities [63]. Nonetheless, various IRBs have differing interpretations of the extent of community engagement and support necessary to proceed with trial enrollment [64]. The logistical complexities in adhering to these requirements and the inconsistent approaches have been identified as obstacles to conducting EFIC research [62]. Notably, only 0.6% of the 63,947 participants in prior EFIC studies voluntarily withdrew or declined to continue participation. The level of acceptability towards the EFIC procedure was usually robust and showed variability contingent on specific circumstances [63]. A critical care sepsis research study demonstrated that WIC increased the number of patients recruited each month from 4 to 10, enabling the study to be completed as planned [48]. Another study, an RCT investigating corticosteroids in TBI, found that the time from injury to randomization decreased by 1.2 h (95% CI 0.7–1.8), and patient recruitment was higher in hospitals where consent was waived than in those that required consent from relatives [65].

A recent report found that EFIC/WIC was applied in 6 of 61 TBI RCTs (10%) [19]. Although these frameworks provide ethical alternatives, it is essential to note that no new medical therapies have been introduced in decades [66]. Over the past twenty years, there has been a growing interest and commitment to studying TBI. Despite this, only a limited number of RCTs and clinical investigations have employed EFIC. Nonetheless, EFIC remains ethically and logistically relevant to TBI research, as it provides a mechanism to include incapacitated patients in time-sensitive trials in an ethical manner. Broader adoption, supported by standardized community engagement frameworks and transparent communication, could facilitate responsible advancement of emergency neurotrauma research [45].

- Proxy-informed consent

Most institutional ethical committees and IRBs in Europe and the USA consider consent from LARs valid and have pragmatically accepted it in accordance with national law [19,67]. This is regarded as a valid surrogate for protecting the rights of incapacitated patients in acute care research and provides protection [24]. Before the intervention, proxy-informed consent is obtained from an individual with legal authority to consent on behalf of the patient to protect the patient from possible misconduct by investigators [68,69,70]. Numerous descriptions in the literature exist of the use of proxies, which may be influenced by different legal frameworks [19,31,69,70,71]. Proxies can include family members, relatives, LARs, Surrogate decision-makers, and, on occasion, independent physicians [19,31,72]. North America, Australia, the European Union, New Zealand, Ethiopia, Chile, China, India, Japan, and South Africa all recognize and approve proxy-informed consent [67,72,73,74]. Its legitimacy is recognized in ethical principles, such as the Helsinki Declaration [75]. However, the patient’s autonomy may be compromised, as proxy permission may not carry the same moral weight as the subject’s consent. The moral underpinning of proxy permission is mainly based on substituted judgment about the study proposal [67,71].

In emergency research settings, alternatives to patient-informed consent are essential in TBI research [17,19]. Nevertheless, several significant challenges stand in the way of obtaining proxy-informed consent. To begin, there is not much time for a consent dialog before an intervention due to the short therapeutic window [73,76,77,78,79]. Numerous studies have identified significant disparities between patients and proxies, leading to the conclusion that proxies often struggle as surrogate decision-makers [80,81,82,83,84,85]. The procedure is further complicated by the fact that proxies are not always readily available or easily accessible, or they may be too stressed to provide a trustworthy proxy-informed consent [86,87,88]. Another barrier is the intricate nature of proxy decision-making. While proxies are generally willing to participate, empirical data indicate that they are not always suitable as surrogate decision-makers [71,77,88,89]. There is skepticism over the proxies’ ability to make decisions that truly reflect patients’ perceptions [88]. In hypothetical settings, significant differences have been reported in decisions made by patients and by proxies [70,90]. According to studies, the false-positive rate of permissions granted by surrogate decision-makers in emergency research might reach 20% [91,92]. Furthermore, Patients’ and their proxies’ agreement on treatment preferences in cases involving coma and brain injury has been shown to range from 57% to 81% [93,94]. The likelihood of this agreement is higher when the patient and proxy have previously discussed what the patient would want in the case of a major sickness or accident [94].

However, given the population’s predisposition to traumatic brain damage (mostly young adults), it seems doubtful that such conversations have occurred frequently. Additionally, TBI can cause cognitive and behavioral changes that make it difficult for patients to communicate their wishes [17]. As a result, proxies are often left to make decisions based on their own beliefs and values. This can be a challenging task, and there is no guarantee that the proxy will make the decision that the patient would have wanted. In emergency and life-threatening situations, most proxies tend to make decisions based on their hopes for the survival of their loved ones rather than considering the likelihood of potential death or disability. This bias in decision-making tends to lean towards possible therapeutic benefits, regardless of how small the chances may be [17,95]. Other factors that may influence decisions include the time sensitivity of the decision [79], perceived study risks or benefits, uncertainty of possible outcomes, the complexity of the patient’s condition, the use of medical terminology, and communication with physicians and nurses [73,96,97,98].

The CENTER-TBI study, conducted across 63 neurotrauma centers in Europe, examined local policies and observed practices regarding informed consent procedures for TBI patients [15,67,74,99]. The study revealed significant variation in accepted informed consent policies and in the actual implementation of consent procedures between and within countries. This variation could stem from various factors, suggesting the absence of clear national or European legislation on the matter or inconsistencies in the knowledge of such legislation among clinicians and researchers. According to the CENTER-TBI study, most IRBs allowed the use of proxy-informed consent (79%) for patients who were mentally incapacitated due to acute conditions. Consent by an independent physician was less commonly permitted (37%). Proxy-informed consent was most utilized in the United Kingdom (96%), Southern Europe (80%), and the Baltic States (76%), whereas its usage was lower in Northern Europe (56%) and Western Europe (49%) [67]. The acceptance or validity of consent by an independent physician varied across the European regions. In intensive care unit (ICU) settings, proxy-informed consent was the most frequent type of consent, followed by patient-informed consent and deferred consent [19,67].

The observed regional variation in proxy consent practices likely reflects differences in legal frameworks, cultural norms, institutional policies, and familiarity with emergency research paradigms rather than inconsistency in ethical standards. Nonetheless, proxy decision-making remains an imperfect surrogate for patient autonomy, as it may not fully reflect patient preferences and is susceptible to emotional and cognitive biases.

Overall, proxy-informed consent has emerged as the most employed alternative, although it was not consistently formalized as a standard policy for patients with DoC. In all cases, however, it was recognized as a legally sanctioned approach under national frameworks. Accordingly, proxy consent should be regarded as a pragmatic and ethically justifiable mechanism to enable research in incapacitated patients, rather than a direct substitute for patient autonomy.

- Deferred Consent in TBI research:

Deferred consent is described as the investigator’s discretionary randomization based on predefined criteria that have been thoroughly discussed during an ethical protocol review [100]. Subsequently, the patient or the legal representative is approached to obtain their informed consent at a later stage [101]. Deferred consent, also known as delayed consent, consent to continued participation, or reconsent from the patient, is commonly used in clinical studies [102]. This procedure is designed to address emergency care situations wherein patients are physically incapable of providing informed consent, as observed in patients with seizures, sepsis, shock, or TBI [103,104]. In the deferred-consent procedure, when the experimental intervention occurs, neither the patient nor their legal representative can provide informed consent due to incapacitation or unavailability [100,101]. Consent for continued participation and data collection is sought only once the patient becomes capable of providing informed consent or a representative becomes available [102]. The World Medical Association issued guidelines in 2013 for “research without prior consent” in emergency cases [34]. Deferred consent may be used if obtaining informed consent from an incapacitated patient is complex and if specific criteria specified in the research ethics committee-approved study protocol are met [34]. These were recognized by the Helsinki Declaration [34] and the International Ethical Guidelines for Human Health-Related Research [105]. Deferred consent is initiated as soon as possible, especially when the study’s intervention has the potential to benefit the participant. It is crucial to note that research can only be conducted with deferred consent, and the risks associated with the intervention must be minimal compared to those of standard therapies [49].

Subsequently, consent must be obtained from the participant or their legal representative as soon as possible after enrolling in the study. Some researchers have proposed implementing a 72 h time limit to prevent unauthorized research activities from being conducted without prior consent. We could not find a foundational basis supporting this time limit in this study [100]. However, the authors relied on a survey conducted among investigators working in the field of TBI to assess the appropriate time limit for obtaining deferred consent. They found that 68% of investigators chose “within 72 h,” and 32% chose more than 72 h [17,49]. If deferred consent is granted, the participant can continue in the trial, and researchers may use previously collected and ongoing data in their analysis [49,61,102]. If obtaining affirmative consent to continue the research becomes unfeasible for reasons unrelated to death, a possibility of patient withdrawal from the study may arise. The conclusion of this outcome is contingent upon the specific parameters and methodologies employed in the study, subject to approval by an ethical oversight committee. Once authorization to continue the study is obtained, previously collected data can be used. Conversely, in scenarios when the continuation of the research is not pursued, the data that has already been gathered can still be utilized as long as patients or their representatives do not exercise their prerogative to reject its use [100].

It has been shown that, in acute care research, most patients enrolled without prior informed consent were willing to provide consent to continue participation [106,107]. A few patients who choose not to continue also refuse consent to use previously collected data [102]. The adoption of deferred consent was supported primarily due to the stress experienced during critical care unit admissions [108]. In such scenarios, the specific procedures are determined by local regulations and the IRB-established criteria. Adhering to local standards is crucial, but there is potential for variations in practice and the use of distinct terminology or descriptions, which may lead to confusion. Researchers need to be aware of this potential issue and possess the flexibility to customize their approach to meet all relevant requirements for using deferred consent for recruiting TBI patients in interventional studies.

Ethical boards have accepted and approved deferred consent in studies conducted in various countries [67,109]. During the COVID-19 pandemic, deferred consent has been used to enroll critically ill patients in essential emergency research to test the effectiveness of therapeutics against the disease. One notable example is the successful use of deferred consent in the REMAP-CAP trial, an international adaptive platform study assessing multiple COVID-19 therapies [110]. Nevertheless, deferred-consent protocols have not been explicitly designed for pandemics, and over the last decade, there has been growing discussion about the ethical appropriateness of this approach [111,112]. Moreover, international variations exist in the legal frameworks governing research in emergency settings. In the USA, deferred consent is allowed under the EFIC code of Federal Regulations, permitting patients to remain in a study even if they do not provide immediate consent upon regaining consciousness. However, some US states have imposed more stringent requirements. Similar disparities are found in European Union member states, with roughly half permitting deferred consent. Canada, parts of Australia, and the UK also allow deferred consent [109].

Despite its legal status, the ethical implications of deferred consent remain debated. Concerns revolve around the perceived infringement on individual autonomy, particularly in “borderline” situations where the urgency of treatment and the patient’s ability to provide prospective consent are less clear [113]. Empirical studies exploring the views of stakeholders involved in the deferred consent process have yielded conflicting results [102,114]. These uncertainties complicate the application of regulatory frameworks, potentially reduce participant recruitment, and hinder the effectiveness of treatment in emergency settings. Therefore, further examination of the ethical acceptability of incorporating deferred consent protocols into research is needed to align with the views of key stakeholders, thereby facilitating the design and conduct of emergency research acceptable to all involved groups.

Deferred consent for enrolling patients in TBI-related RCTs was rarely acknowledged as a viable option, accounting for only 13% of cases [19]. Another CENTER-TBI study reported that 45 centers across 10 countries employed this alternative consent approach. However, only 15 centers, representing 26% of the total, actively used it [67]. Several factors contribute to this variation. Firstly, although national laws may acknowledge deferred consent, it is possible that the respective IRBs did not formally authorize its use within the CENTER-TBI project [67].

Additionally, it is essential to recognize that deferred consent presents ethical complexities, and its acceptance varies among IRBs, healthcare providers, patients, and their representatives. Moreover, although deferred consent was considered valid, it might not have been mandatory, as proxy or independent physician consent was obtained. Lastly, many researchers may not have been aware of the option to employ deferred consent.

Table 1 shows the pros and cons of alternate consent strategies in clinical and translational traumatic brain injury research.

**Figure 3:**
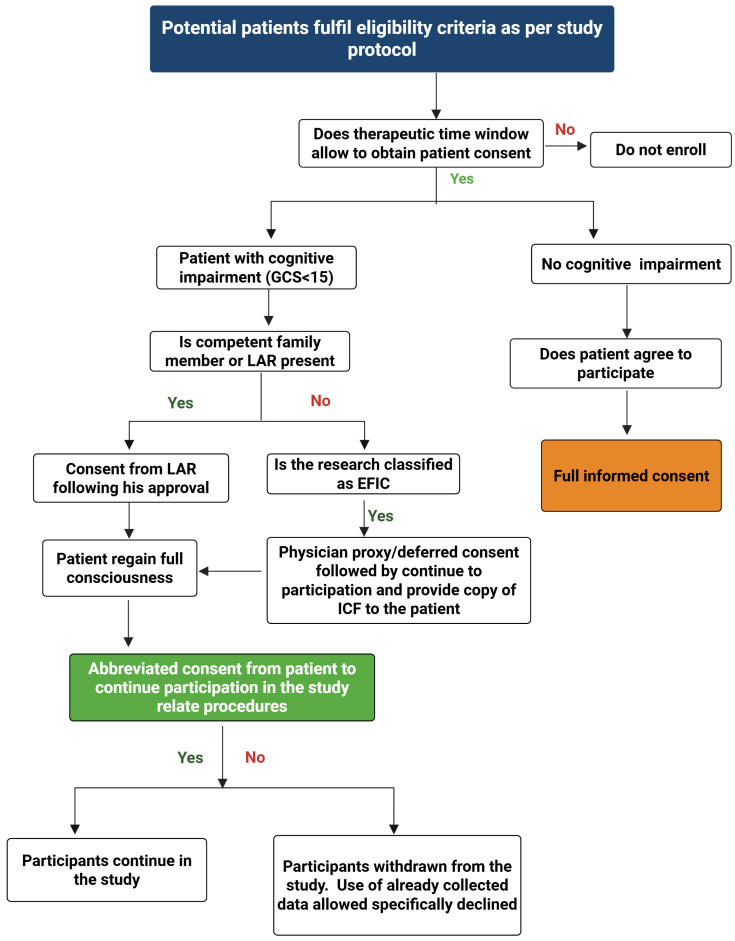
Flow chart for the selection of appropriate informed consent procedures for interventional clinical and translational TBI research: (EFIC: exception to informed consent; GCS: Glasgow Coma Score; ICF: informed consent form; LAR: legally authorized representative).

The challenges associated with obtaining consent before initiating TBI emergency interventional research can result in missed research opportunities if alternative consent methods are not implemented. To improve the utilization and effectiveness of consent procedures in TBI research, it is essential to navigate a structured decision-making process. Researchers should initially assess whether the therapeutic time window allows an informed consent procedure. If time permits, they should then determine if obtaining valid patient or proxy-informed consent is feasible before the intervention within that time frame. If both are impossible, the patient’s wishes regarding study participation may be indeterminable. In such cases, researchers should explore alternative procedures, such as deferred consent/Physician proxy consent, an exception from informed consent, or a waiver of informed consent, primarily depending on local laws and specific study details. All procedural decisions regarding informed consent must comply with applicable ethical and legal requirements. We recommend a personalized approach for selecting the appropriate informed consent procedure. This approach is designed to guide future investigators and researchers conducting clinical and translational studies in TBI research. Figure 3 shows a flow chart for the selection of appropriate informed consent procedures for interventional clinical and translational TBI research.

### 3.6. Regulatory Barriers: IRBs and the Ethical Framework of TBI Research

A Research Ethics Committee or IRB aims to evaluate research procedures to ensure they are in accordance with ethical standards and national laws. IRBs play a crucial role in safeguarding the dignity, rights, and well-being of research participants, and their formal endorsement is mandatory before commencing a clinical study [115]. Moreover, advancements in medical science and technology are progressing rapidly. As a result, the number of clinical trials involving multiple sites and diverse cultural settings is growing. However, this increase in the number of trials has created challenges and discrepancies in IRB decisions [14]. Therefore, a comprehensive understanding of the challenges and obstacles posed by regulations is crucial to maintaining consistent patient safety in trauma and TBI research. A lack of information on regulatory prerequisites frequently leads research teams to have an incomplete understanding of the intended objectives of particular laws, regulations, and policies [31]. Some scholars from ethical and legal backgrounds argue that the risks to research participants are minimal, while the burden imposed by IRB review can be significant [116]. Additionally, there is a range of interpretations and applications of regulations among different IRBs [117].

Despite several global models designed to promote harmonization of ethical standards, IRBs are influenced by national legislation and regulations, which shape their organizational structure and functions to accommodate local needs [118]. This results in differences in the approval process for research protocols submitted to IRBs, potentially complicating the execution of international research [119]. Addressing differences in IRB processes has become increasingly crucial as collaborative TBI research activities have expanded [21]. The lack of harmonization in research and IRB processes leads to an ethical review framework and difficulties in obtaining informed consent from participants, resulting in inefficiencies in prospective observational and interventional TBI studies [120]. Therefore, it is widely recognized that achieving harmonization is desirable as it can improve the quality and efficiency of healthcare research in various ways [121]. This includes enhancing scientific validity, enrolling patients, optimizing data management, selecting outcome variables that can be generalized, and minimizing resource waste caused by inefficiencies [122]. Although efforts are underway to adopt research regulations, the complexity of regulations rooted in customs, culture, ethics, religion, and other beliefs poses challenges.

The CENTRE-TBI study, involving 18 European countries and 66 centers, reported significant variation in IRB procedures within and between countries, indicating a lack of standardized legislation and inconsistent implementation of regulations [118]. Some countries require central IRB approval to start a study, while others require an additional IRB review at the participating site. Additionally, there were differences in the number of review rounds, the time required for IRB approval, and the nature of IRB questions and comments. Importantly, not all IRBs categorize the study as observational or interventional, indicating differences in interpretation of the study’s protocols [118]. These variations may be influenced by IRBs’ different interpretations of study procedures as either standard of care or not.

The time required to obtain IRB approval to conduct a study after submitting a protocol varies significantly, from months to years [21,123]. The difference in duration between central and primary local IRB approval is not solely based on the number of review rounds but can be influenced by factors such as the reason for additional review, the nature of the review process, the workload involved, and the efficiency of both the IRB and research team [124]. Additionally, in some countries, the time spent responding to IRB questions affects the duration of the approval process. On the other hand, some countries attributed the delay in the approval process to the time required for IRB evaluation [118]. The exact causes of these delays are not clear yet. However, IRBs often seek explanations regarding research procedures. Therefore, attempts should be made to reduce delays by investigators and researchers by responding quickly and setting strict timetables for IRB approval.

Moreover, the need for IRB reviews after primary central IRB approval can lead to avoidable delays, further impeding research progress. Unnecessary delays in obtaining IRB approval have a detrimental overall influence, including hindering study initiation, causing frustration among researchers/academicians, and potentially compromising the quality and applicability of study findings [21,125]. For instance, the approval for the study of prehospital tranexamic acid in trauma patients took around five years [59].

**Figure 4:**
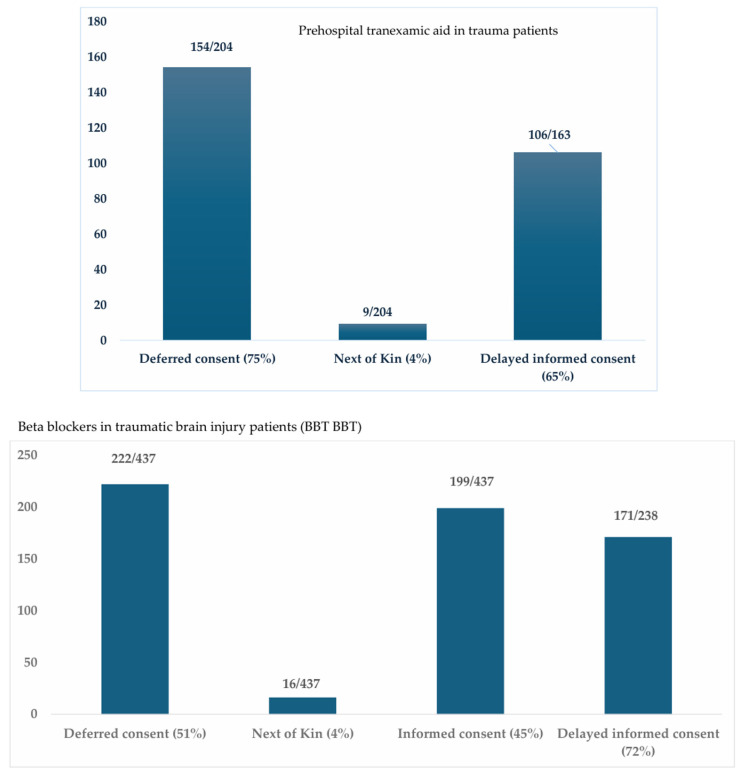
Type and percentage of consent in studies involving trauma patients. For the tranexamic acid in trauma study [66], 154 deferred consent and 9 next of kin consent = 163/204 patients (79.9%), of which 106 were able to complete the delayed informed consent (65%). Total of 41/204 patients signed informed consent on admission (20.1%). For the beta blockers in TBI study [67], 222 deferred consent and 16 next of kin consent = 238/437 patients (54.5%), of which 171 were able to complete the delayed informed consent (72%). Total of 199/437 patients signed informed consent on admission (45.5%).

Figure 4 shows the percentage of each type of consent in two of our studies during the recruitment of trauma patients [59,60].

Moreover, multiple rounds of review may introduce bias and compromise the study’s scientific integrity [125]. Implementing reforms can alleviate burdens while maintaining participant safety, aligning with the 2017 Department of Health and Human Services (DHHS) amendment to the Common Rule [126]. The amendment aims to streamline processes and enhance protection by mandating that most multisite trials be reviewed by a single, central IRB rather than multiple parallel IRBs. A central IRB serves as the examining body for assessing all sites involved in one or more multisite studies. The amendment also aims to reduce the number of regulations that allow IRBs to prioritize higher-risk studies. It introduces exemptions for low-risk studies, reducing the need for IRB review. In some cases, ongoing IRB review may no longer be necessary. Moreover, it allows research participants to consent to use their information and biological samples in studies [127].

However, continued skepticism about using a single or central review for multisite trials stems from worries about the importance of local issues, responsibility, and liability [120,123]. There are concerns about losing control of the review process, doubts about the quality of reviews undertaken by other IRBs, and practical difficulties such as cost-sharing. There is insufficient data to compare a single or central review to local site reviews regarding review quality, satisfaction, resource utilization, or efficiency.

Nevertheless, simplifying the review process for site studies, reducing IRB reviews of low-risk studies, and giving research participants the option to provide broad consent for future studies have been recommended as potential measures. Additionally, making publicly available checklists that outline the criteria for IRB review can ensure transparency and consistency in review processes across studies and IRBs. Unnecessarily long and tedious research ethics approval processes undermine the perceived efficiency of the process, leading to greater disappointment among academics.

While IRB oversight has evolved over the past four decades, the rules, procedures, and goals have not kept pace with advances in research methods and opportunities. Data, creativity, regulatory flexibility, and ongoing dialog are crucial in addressing this disparity. These efforts are crucial for advancing clinical research, maintaining public trust, protecting research participants, and upholding fundamental ethical principles. Table 2 summarizes ethical insights and regulatory recommendations for researchers in TBI-related clinical and translational studies.

### 3.7. Summary, Reflections, Anticipations, and Recommendations

The complexity of healthcare settings, the urgency of situations, and the seriousness of patient injuries create significant difficulties in conducting clinical and translational research on TBI patients in trauma settings [61]. These challenges hinder the potential for life-saving research focused on advancing pharmacotherapy, testing medical devices, and developing technologies to enhance patient outcomes and recovery [27]. Regulatory measures designed to safeguard research participants can impede the progress of essential scientific innovations required to treat critically ill and injured individuals with TBI. Striking a balance between these regulatory imperatives in emergencies is complex. Researchers must address public apprehension, streamline regulatory compliance, and ultimately expand their application to enable timely, ethically sound research that advances emergency care and improves patient outcomes. Steps to alleviate limitations and facilitate the use of EFIC are presented in Table 3 [19,24,33,71,73,79,118,126]. Informed consent in emergency neurotrauma research should more appropriately be conceptualized as a dynamic and iterative process rather than a discrete event.

Limitations: the lack of quality assessment of studies is a key limitation in this review.

## 4. Conclusions

Research involving patients with TBI entails distinct ethical complexities that require careful consideration. Investigators must be familiar with applicable local and international regulations and integrate these frameworks into study design and conduct.

Obtaining informed consent is sometimes not feasible in the TBI settings due to a limited time-frame, impaired patient capacity, or a lack of surrogate decision-makers. Although alternative approaches such as proxy or deferred consent are ethically acceptable, they remain underutilized.

Consent processes should therefore balance ethical rigor with clinical pragmatism. Recognizing their inherent limitations while transparently addressing methodological implications is essential in neurotrauma research.

This review calls for clarifying terminology, modifying arbitrary regulatory restrictions on research, and streamlining the Common Rule, while safeguarding patient rights.

Scientists should share their innovative solutions to strike a balance between ethical considerations and minimizing research barriers.

## Figures and Tables

**Table 1:** Pros and Cons of Alternate Consent Strategies in Clinical and Translational Traumatic Brain Injury Research.

Modalities	Advantages	Disadvantages
**Proxy-Informed consent**	**Timely Enrollment:** Allows for immediate enrollment of stroke patients who are unable to provide consent themselves, ensuring prompt treatment. **Increased Participation:** Expands the pool of eligible participants by including those with reduced decision-making capacity. **Ethical Consideration:** Offers a solution when obtaining informed consent from the patient is not possible due to their condition. **Potential Life-Saving and Scientific Benefits:** Enables access to potentially lifesaving or life-improving treatments during the critical early phase of TBI, which may enhance scientific insights and therapeutic treatments and medical knowledge. **Research Continuity:** Facilitates the continuation of emergency TBI trials and clinical studies, ensuring a robust and comprehensive study population.	**Ethical Concerns:** Raises ethical questions about the authenticity of informed consent when obtained from a proxy. **Patient’s Autonomy Issues:** May not fully respect the autonomy of the patient, as their wishes are not directly considered. **Proxy Understanding:** Relies on the proxy’s understanding of the patient’s preferences and may not accurately reflect the patient’s wishes. **Legal Challenges:** Legal regulations and guidelines regarding proxy consent may vary, creating challenges for standardization. **Consent Quality:** The quality and accuracy of proxy-provided consent may vary, potentially affecting the validity of the study results.
**Deferred Consent**	**Ethical Inclusion:** Enables the inclusion of patients in emergency stroke trials who would otherwise be excluded due to the urgency of the situation. **Research Continuation:** Allows researchers to initiate treatment promptly, potentially improving outcomes. **Community Engagement:** Fosters engagement and awareness of clinical research within the community.	**Ethical Concerns:** Raises ethical questions regarding autonomy and informed consent. **Varying Legal Frameworks:** Deferred consent legality varies by region and may not be permitted in some regions. **Stakeholder Conflicts:** Conflicting stakeholder views on the ethical acceptability of deferred consent. **Data Use Uncertainty:** Data collected under deferred consent may not be used if consent to continued participation is not obtained.
**Exception to Informed Consent or Waiver of Consent**	**Rapid Intervention:** Allows immediate intervention for TBI patients leading to the inclusion of a wider range of participants, including those who are unconscious or lack capacity or who may not have a legal representative or proxy readily available. **Real-World Conditions:** Reflects real-world emergencies, providing more accurate data for clinical research. **Timely Research:** Accelerates the research process by removing the need for extensive consent procedures. **Efficiency:** Reduces administrative burdens, paperwork, and delays associated with obtaining informed consent.	**Ethical Concerns:** Raises ethical questions about respecting participants’ autonomy and informed decision-making. **Informed Decision:** Participants may not have the opportunity to fully understand the research and its risks before participating. **Safety Concerns:** Potential for exposing participants to unproven treatments without their explicit consent. **Regulatory Challenges:** Navigating regulatory approval and satisfying ethical review boards can be complex and time-consuming. **Community Perception:** May lead to negative public perception or skepticism about the research. **Limited Data:** Limited or missing data on the long-term consent preferences of incapacitated participants.

**Table 2:** Ethical Insights and Regulatory Recommendations for Researchers in TBI-Related Clinical and Translational Studies.

Ethical Dilemmas Clinical and Translational Research Related to TBI	Key Considerations for Academicians/Researchers/Physicians
Autonomy and informed consent for patients with cognitive impairment	Respect for autonomy is the foundation of voluntary and informed consent. TBI often involves the study of conditions that lead to cognitive impairment, making informed consent challenging. Rather than excluding patients with decisional incapacity from research, alternative approaches should be considered. When a participant lacks decision-making capacity, a legally authorized representative/surrogates can make decisions on their behalf. The 2017 Common Rule amendment allows surrogates for medical decision-making to make decisions for clinical research. Efforts should be made to protect participants with cognitive impairments while minimizing barriers to research in this population. Such as IRBs granting EFIC to ensure swift initiation of research-related procedures while maintaining ethical standards.
Time sensitivity nature of TBI Research	The time-sensitive nature of TBI research underscores the critical importance of rapid diagnosis, early intervention, and ongoing advancements in the field to improve patient outcomes. The ethical use of EFIC with oversight from IRBs ensures that research can be conducted promptly while safeguarding the rights and well-being of participants. This approach strikes a balance between the urgent medical needs (Therapeutic window) of TBI patients and the ethical principles of research involving human subjects.
Balancing benefits and risks in TBI-related clinical studies	Substituted consent is crucial for preserving participant autonomy in TBI clinical trials, but it is not enough to ensure ethical research. Assessing risks and benefits in TBI studies is challenging because patients cannot report their subjective experiences, making risks unknown and benefits difficult to evaluate. Outcome assessment tools often rely on researcher-defined criteria rather than the perspectives of TBI patients and caregivers. There is a lack of consensus on the level of treatment intensity and assessing the quality of life for TBI patients, making defining a desirable outcome a challenge. Trials involving drugs for individuals with disorders of consciousness must carefully evaluate potential risks and benefits, including side effects and contributions to treatment development. It is important to communicate unknown psychological effects of interventions, maintain clinical equipoise, and balance benefits and risks, with local IRBs assessing fairness and appropriateness.
Alternative consent procedures	Ethical and Legal Compliance: Ensure compliance with national and international ethical guidelines and legal regulations regarding alternative consent procedures. Institutional Review Board (IRB) Approval: IRB approval for using deferred, exception, or waiver of consent, and adhere to IRB recommendations. Patient or Proxy Involvement: If prior informed consent is not feasible, consider whether patients or their proxies can still be involved in the decision-making process. Identification of the most appropriate consent process, i.e., deferred, proxy or EFIC for recruitment of TBI patients. Determination of whether the therapeutic time window allows for informed consent or if it is infeasible. Engagement with the local community to understand their perceptions of the research and its consent process. and adherence to local laws and regulations regarding consent procedures. Accurate document the consent process and ensure it aligns with chosen alternative methods and collection of data to evaluate the quality, satisfaction, resource use, and efficiency of alternative consent procedures. Clear communication of the procedures used and respect for the patients’ right to refuse study participation or data use to maintain transparency. Continuously monitoring and adherence to applicable legislation and regulations during the research. Strive for high-quality research by minimizing the potential biases introduced by multiple reviews.
Harmonization of IRB approval for the TBI research	IRB Variability in Decision-Making: IRBs play a crucial role in ensuring research ethics and participant well-being, but there is significant variability in their decision-making, both within and between countries, which can lead to inconsistencies and delays in research approvals. Differences in national legislation and regulations shape the organizational structure and functioning of IRBs, resulting in a lack of regulatory harmonization. This lack of alignment complicates international research, including studies related to TBI. IRB review durations vary widely, with approval timelines ranging from one day to one year. Factors contributing to these delays include the number of review rounds, response times, evaluation processes, and workload. Collaboration between researchers and IRBs is essential to streamline the review process and minimize avoidable delays in research progress. Delays can negatively impact study initiation, researchers’ morale, and the quality of study findings. Skepticism exists regarding the use of single or central review for multisite trials, with concerns about the importance of local issues, responsibility, and liability. There is also uncertainty about the quality of reviews by other IRBs and practical challenges like cost-sharing. Data comparing single or central reviews to local site reviews are lacking, making it difficult to assess their quality, efficiency, and resource utilization.

**Table 3:** Steps to alleviate the limitations and facilitate the use of EFIC.

Primary Steps	Secondary Steps
Enhance community consultation and public disclosure.	Utilize multiple platforms, including social media campaigns, online surveys, and educational displays at public venues.
Actively listen to community concerns, address misconceptions (e.g., fear of receiving less effective treatment), and share the feedback with the IRB.	Establish clear, accessible options such as bracelets or online registries for individuals to decline participation in EFIC studies, thereby upholding personal autonomy while facilitating ethically responsible research.
Streamline regulatory and ethical review.	Implementing a centralized IRB review system for multi-center trials/studies can improve efficiency and uniformity, avoiding redundant reviews across sites.
Collaborate with regulatory authorities (like the FDA) to establish clear, harmonized guidelines for EFIC implementation, especially for procedures like timely engagement with LARs.	Develop standardized, evidence-based templates for community consultation, public disclosure materials, and consent forms that can be customized for site-specific needs.
Utilize established research networks to share data, resources, and best practices, fostering quality improvement for future studies.	Commit to publicly disclosing study results, regardless of the outcome, to maintain trust with the community and uphold the ethical obligation to advance medical knowledge. Align research protocols with existing clinical workflows and procedures to encourage engagement from frontline healthcare providers.
Offer consistent training for research staff and clinicians on EFIC procedures and the ethical foundation to ensure protocol adherence and transparent communication with the families.	Inform patients or their LAR about study enrollment as soon as possible and seek prospective consent for continued participation.

## Data Availability

No new data created or analyzed.

## Supplementary Materials

The following supporting information can be downloaded at: https://www.mdpi.com/article/10.3390/neurosci7030051/s1. Preferred Reporting Items for Systematic reviews and Meta-Analyses extension for Scoping Reviews (PRISMA-ScR) Checklist.
